# Emerging Nipah Virus With Pandemic Potential and High Mortality Rates: Is the Scientific Community Learning From Former Pandemics?

**DOI:** 10.1002/rmv.70028

**Published:** 2025-03-05

**Authors:** Doris Klingelhöfer, Markus Braun, Christina A. Naser, Dörthe Brüggmann, David A. Groneberg

**Affiliations:** ^1^ Institute of Occupational Social and Environmental Medicine Goethe University Frankfurt Frankfurt Germany; ^2^ Paul Ehrlich Institute Langen Germany

**Keywords:** COVID‐19, emerging infectious diseases, henipa virus, mortality rate, mpox, pandemic

## Abstract

As Nipah virus (NiV) infection is characterised by a possible pandemic risk, being currently limited to a small but deadly belt, the attention of other countries is essential. It has often been pointed out that NiV is an under‐researched virus with a high‐risk potential. This study aimed to show the global research history and status quo based on analyses of various chronological and geographical parameters, including socioeconomic characteristics and research funding. For this purpose, advanced analysis methods and visualisation techniques were applied, such as density equalisation mapping and cluster analysis. The correlation between the number of articles on NiV and the economic strength or intensity of financing per country is significant. However, the comparatively low scientific commitment of countries that are usually among the major players in global scientific publications and the declining scientific interest in NiV research combined with the prevailing knowledge gaps in NiV infectiology in conjunction with the risk of NiV spreading to other areas is extremely threatening. Research on previous viruses such as Corona and mpox shows an equally short‐term interest, which has led to an insufficiently prepared situation in the run‐up to outbreaks, making it hard to find quick and effective solutions. As often said, the NiV infection belt is small but deadly, but global travel and trade increase the risk of spreading. The scientific community worldwide must be prepared for the possible spread of infections that pose a pandemic risk.

## 1 Introduction

The Nipah virus (NiV) is an emerging zoonotic virus from the paramyxovirus family and a highly pathogenic single‐stranded RNA virus that is endemic in Southeast Asia and the western Pacific. Without targeted countermeasures, NiV could trigger a pandemic under certain circumstances [1, 2]. Although the infection is currently limited to a small but deadly belt, the urgent call for preparedness in other countries is imperative. The spreading increases with the transmission factor R0, the tendency to mutate, the transmission from human to human or animal to human, and the lack of adaptation in humans [3]. Not only are the neighbouring countries of countries with NiV outbreaks at risk, but there is a global threat. Since globalisation not only facilitates contact with infected animals or other trade commodities to transmit the disease but also enables human‐to‐human infection, the area of NiV occurrence may increase [4, 5]. In addition, the risk of transmission to humans increases due to the wide range of bats, especially in previously unaffected areas where there is a lack of experience with testing, detection, and treatment [6].

After five outbreaks of NiV infections, some of which were sporadic and only affected one or two infected people, the last outbreak in India to date occurred in September 2023. Only in the first case is the source of infection unknown. The later infections could be traced back to human contact with family members or medical staff [4].

NiV is named after Sungai Nipah, a Malaysian village where the virus was first detected in farmers in 1998 [7]. It is closely related to the Hendra virus, which also belongs to the paramyxoviruses [8]. NiV encodes two glycoproteins, the receptor‐binding protein G and the fusion protein F [9]. Two main genotypes of NiV have been described. Clade I: NiV‐B was detected in India and Bangladesh. Clade II NiV‐M was reported in Malaysia and Cambodia [10]. The Bangladesh type, in particular, is more virulent. It has been shown to have higher pathogenicity and more severe clinical manifestations [11, 12]. So far, Malaysia, Singapore, Bangladesh, India, and the Philippines have reported one or more outbreaks [10]. The natural reservoir hosts of NiV are mainly four species of flying foxes of the genus *Pteropus*, also known as fruit bats [13, 14]. These hosts have been found close to human dwellings and have even entered them. The most common intermediate hosts are pigs [15]. As a zoonotic virus, NiV is particularly difficult to combat because the entry receptor EFNB2 is highly conserved in mammals [16]. In some communities in Nepal, flying foxes are consumed as bushmeat, indicating the associated risk [17]. NiV can also be transmitted through body fluids, contaminated food with urine, saliva, or excretions from infected animals [4, 18].

Depending on the strain, different symptoms occur [19]. The strain from Bangladesh frequently causes severe respiratory diseases and atypical pneumonia, while the Malaysian strain is more associated with neurological symptoms such as severe encephalitis [11, 20, 21, 22]. Symptoms such as headache, vomiting, myalgia, disorientation, fever, cough, and shortness of breath are typical of the initial phase of NiV infection. The case‐fatality rate (CFR) is between 40% and 70% and more [4], which is much higher than that of SARS‐COV‐2, which was between 1.7% and 39% at the beginning of 2020 and fell drastically to below 0.3% in July/August 2023 [23]. The CFR of NiV varies depending on the strain and regional characteristics such as surveillance, clinical management, or hygiene, for example, wearing masks or gloves [24]. It is therefore classified as a biosafety level 4 (BSL‐4) pathogen for pathogens in the highest risk group, such as Ebola and Lassa, which requires the highest safety precautions in laboratories. Although their number is increasing, in 2023, there were only 51 BSL‐4 laboratories in 27 countries worldwide [25]. Although vaccine and treatment development is being focused on in recent clinical trials [26], no vaccine or therapeutics are yet available for humans or animals as there are no other treatment options. In humans, primary supportive treatment is provided [24], focussing on severe neurologic and respiratory complications [4, 27].

There are many unknown aspects of NiV. The virus is not yet fully understood, and knowledge about its transmission and development is still insufficient. The same applies to virus circulation in bats and the spread to domestic animals [10].

In addition to the high fatality rates and pandemic risk, NiV infection can cause significant economic losses, especially for farmers in the affected low‐income countries, as their livestock is at risk [24]. The close clustering of outbreaks often leads to a community crisis, as the affected persons know each other [28]. The illness of several people in a household affects the family income both in the short and long term. The need for cost‐intensive healthcare combined with sometimes forced sales of income‐generating assets also contributes to poverty. In addition, there are indirect costs due to the loss of income and educational opportunities [29, 30].

As there is neither a vaccine nor antiviral treatment, control measures that should extend to isolation and contact tracing are necessary but also cost‐intensive and, therefore, usually limited by the socio‐economic basis of the affected countries [31]. Research approaches must consider these conditions in conjunction with regional epidemiological and logistical characteristics [32].

Given all these implications, the World Health Organization (WHO) has pointed out that research on NiV urgently needs to be accelerated [24]. Undoubtedly, the global scientific communities have already responded, but to what extent and where is the research mainly conducted? Is the research also relevant and appropriate regarding exposure and disease risk? To answer these questions, this study aims to provide a background portfolio of global research patterns for all stakeholders, from individual scientists to decision‐makers and funders. To date, there is no consistent study on NiV research from a socio‐economic perspective that also includes financial aspects and refers to previous pandemics.

## 2 Methods

The analysis and visualisation methodology used for this study is based on the established procedures of the bibliometric platform “New Quality and Quantity Indices in Science” (NewQIS) [33, 34]. All NewQIS studies use the Web of Science (WoS) Core Collection as the default database for querying the metadata of the publications. Therefore, WoS is also used to search and retrieve data.

The term “Nipah*” was searched in the title and abstract of the publication, with the terms “virus*” or “outbreak*” applied in the WoS search tool TOPIC (title, abstract, keywords) to exclude articles about the Nipah fruit, the Nipah worm, or the similarly named indigenous writing materials made from palm leaves. The asterisk serves as a placeholder and enables the inclusion of various subsequent parts of speech, such as the plural form of the term. Once the metadata of the publications found in this way had been recorded, they were stored in an MS Access database, sorted, normalised, and processed before being analysed and interpreted.

Using established tools of the NewQIS methodology, global publication patterns were analysed from different perspectives, ranging from chronological and geographical to epidemiological and socio‐economic analyses. In addition, the visualisation technique of map cartograms was introduced in NewQIS to present global values clearly and easily understandable. These “Density Equalising Map Projections” (DEMPs) are generated by an algorithm developed by Gastner and Newman that distorts the sizes of all countries according to the analysed parameter [35]. This tool is used here to analyse and evaluate the countries from which the publications on NiV originate. In addition to the publication volume, the number of citations received, the citation rate, the ratios of the number of articles on NiV, and a demographic (population size) and economic value (gross domestic product/GDP) [36] were mapped as DEMP. In addition, analyses of keywords and WoS categories were carried out and evaluated, showing the thematic focus of NiV research. Some of these were visualised with the help of VOSviewer [37]. Thematic clusters were formed. To expand the significance of the evaluation of the research priorities, the WoS categories were analysed according to their number of assignments, their development over time, measured by the assignments in 4‐year intervals, and their assignments to the country of origin. The cooperation networks of NIV research were also part of this analysis at the country and the institutional level. The evaluation requires the identification of the most publishing institutions. To obtain a valid result of the institutional ranking, the extremely varying designations of the publishing institutions must be standardized, which requires a careful approach. The resulting network diagrams show the results of the scientific partnership on NiV. The status of single authorship of the publishing countries was determined, as well as the percentage share of first and co‐authorship in the cooperation articles. Another analysis included in this study concerned international funding. The most funding countries and organizations were identified. The analysis of research funding did not include financial funding for the publication fees, for example, open access publication fund DEAL (Germany) [38]. Only the funding mentioned by the authors in the manuscript was taken into account. Linear regressions were carried out, and Spearman correlations were calculated to assess a possible association between the number of articles on NiV per country and the number of approved funds and economic strength measured by GDP. The residuals were then evaluated according to the positive or negative deviation of the respective country from the regression line.

### 2.1 Limitations

The difficulty of any bibliometric study is to achieve representativeness through the most complete and least erroneous data possible, which can only be guaranteed by an advanced search strategy. By limiting the main term search (Nipah) to the title and abstract and additionally, including virus‐related terms in the authors' keywords, this study ensures that the reference to the research topic is guaranteed and that the majority of articles are found. In addition, a manual check was carried out to validate the resulting database. In this context, the limitations of WoS, which do not allow all journals to be indexed and thus included in the study, must also be considered when interpreting the results. The often‐discussed English bias of the included indices must also be taken into account.

A further methodological limitation exists in the case of publications with collaborating authors. In this bibliometric analysis at the country or institutional level, no distinction can be made with regard to the degree of individual participation. Therefore, all participating institutions or countries are treated equally.

Furthermore, the significance of the citation parameters must be viewed critically. The superiority in this respect indicates greater recognition in the scientific community but does not allow any concrete and unambiguous statements to be made about the quality of the research. The figures are also susceptible to a variety of distortions, such as self‐citations or misquotations.

## 3 Results

The WoS core collection identified 1431 publications (*n*) on the topic of NiV. Of these, 955 publications were original articles (66.74%). As many as 14.95% (*n* = 214) were published as reviews. The other types include editorial material, letters, conference summaries, news, conference reports, and corrections. Almost 98% of all publications were written in English.

### 3.1 Research Foci

**FIGURE 1:**
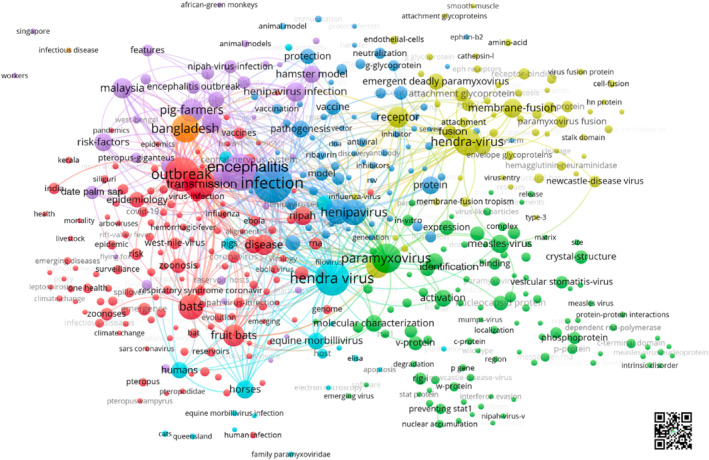
Cluster analyses of keywords (threshold cluster: 5 occurrences, threshold connections: 10 occurrences). Cluster coding: Blue: Infection; Violet: Encephalitis, Malaysia; Red: Transmission, outbreak; Green: Identification, characterisation of the virus; Yellow: Gene expression, protein fusion; Light blue: Hosts, Orange: Bangladesh. QR code: Interactive online version.

The analysis of the keywords in the articles revealed various thematic clusters. The most frequently used groups are clustered around the terms “outbreak”, “encephalitis” and “hendra virus” and reflect the foci on transmission and outbreaks (red cluster), infection, pathogenesis, vaccination (blue cluster), encephalitis in Malaysia (purple cluster), virus identification and characterisation (green cluster), protein fusion, and gene expression (yellow cluster) as well as the smaller groups of analysis on hosts (light blue cluster) and the focus on Bangladesh NiV (orange cluster) (Figure 1).

The evaluation of the WoS categories shows the most frequently assigned research areas. The categories with more than 100 assignments are as follows: *Virology* (*n* = 414), *Infectious Diseases* (*n* = 198), *Microbiology* (*n* = 151*), Science & Technology—Other Topics* (*n* = 121), *Immunology* (*n* = 104) and *Public Health, Environment and Occupational Safety* (*n* = 101).

### 3.2 Development Over Time

The first articles that met the search requirements were published in 1999, but only in single‐digit numbers. Double‐digit annual figures have been reached since 2000, with the first peak in 2009 with *n* = 70 articles. This was followed by a period of undulating development, which has been characterised by a sharper increase since 2018. The previous peak was reached in 2020 (*n* = 2020), only to fall again afterwards. The annual trend in citation figures developed inversely to the publication figures, starting with a high value after the first year of publication with more than 2000 citations (c). Subsequently, a downward trend in the number of citations can be observed, also with an upward and downward course. They reached their highest value to date in 2012 with *c* = 3375. Later, another interim peak was reached, interrupting the steady decline since then (2019, *c* = 2379).

**FIGURE 2:**
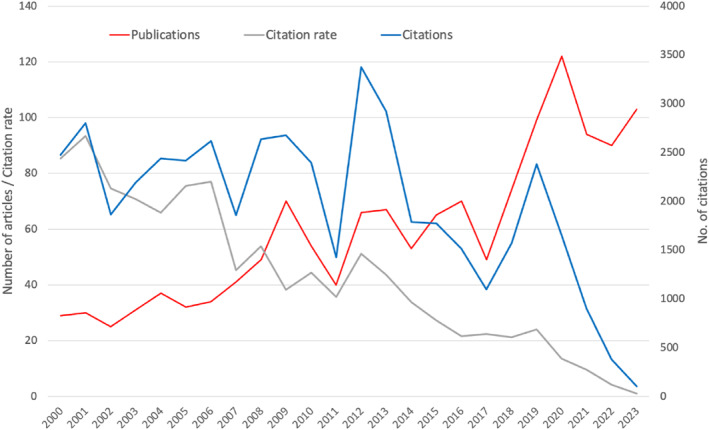
Development from 2000 to 2023. Number of publications and citation rate (primary *y*‐axis), number of citations (secondary *y*‐axis).

This generally opposing trend in annual publication and citation figures is reflected in the development of the citation rate with its steadily decreasing trajectory (Figure 2).

Table 1 lists the ten most frequently cited publications indicating the influence of USA, Malaysian, German, British, Chinese, and Australian scientists in NiV research. These top 10 were all published up to 2013. The most frequently cited article comes from the journal Science from the year 2000 and deals with the specification of NiV as a deadly paramyxovirus [39]. Malaysia, where NiV was first described, has thus found its place in the top 10.

**TABLE 1:** Most cited articles about NiV up to the evaluation date.

Authors (Country of origin of first author)	Year	Citations	Title	Journal
Chua et al. (Malaysia)	2000	868	Nipah virus: A recently emergent deadly paramyxovirus	Science
Daszak, Cunningham, and Hyatt (USA)	2001	625	Anthropogenic environmental change and the emergence of infectious diseases in wildlife	Acta Tropica
Chua et al. (Malaysia)	1999	506	Fatal encephalitis due to Nipah virus among pig‐farmers in Malaysia	Lancet
Drexler et al. (Germany)	2012	461	Bats host major mammalian paramyxoviruses	Nature Communications
Liu et al. (USA)	2013	440	Interferon‐Inducible Cholesterol‐25‐Hydroxylase Broadly Inhibits Viral Entry by Production of 25‐Hydroxycholesterol	Immunity
Chua et al. (Malaysia)	2002	423	Isolation of Nipah virus from Malaysian Island flying‐foxes	Microbes and infections
Zhang et al. (China)	2013	397	Comparative Analysis of Bat Genomes Provides Insight into the Evolution of Flight and Immunity	Science
Weiss and McMichael (UK)	2004	372	Social and environmental risk factors in the emergence of infectious diseases	Nature Medicine
Johara et al. (Australia)	2001	359	Nipah virus infection in bats (order Chiroptera) in peninsular Malaysia	Emerging Infectious diseases
Solomon et al. (UK)	2003	355	Origin and evolution of Japanese encephalitis virus in southeast Asia	Journal of Virology

### 3.3 Country Patterns

Out of the total number of publications, *n* = 1384 (96.72%) could be assigned to a country of origin that builds the basis for all geographical analyses.

**FIGURE 3:**
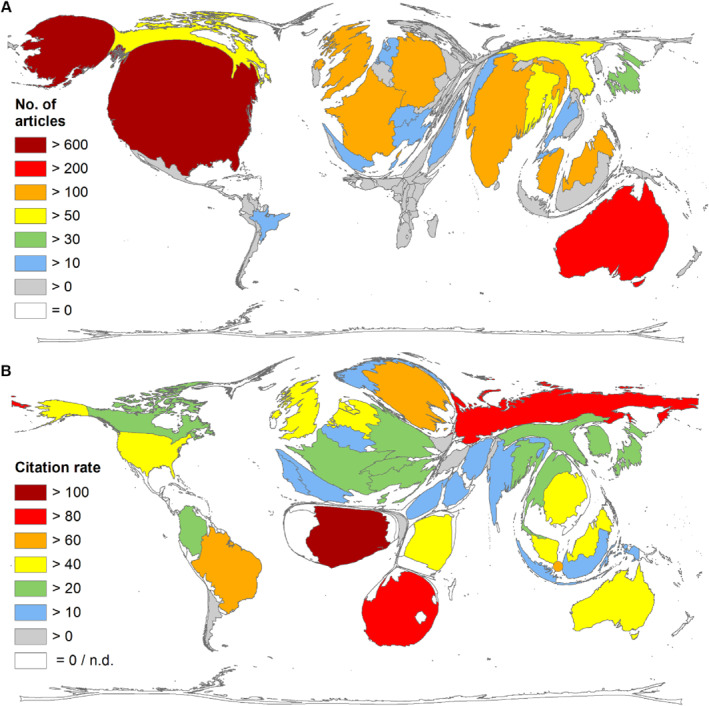
Countries' pattern on NiV research. (A) Number of articles per country. The five most publishing countries: USA, Australia, India, Malaysia, France. (B) Citation rate (threshold: 5 publications on NiV). The five countries with the highest citation rate: Ghana, Russia, South Africa, Sweden, Singapore (Added as a point. It could not be shown as an enlarged country due to the physical limitations of DEMP technology).

The country with the most publications on NiV is the USA (*n* = 655), followed by Australia (*n* = 227), which published only around a third of US publications. In third place is India (*n* = 159), followed by Malaysia (*n* = 144) and France (*n* = 123) (Figure 3A).

**FIGURE 4:**
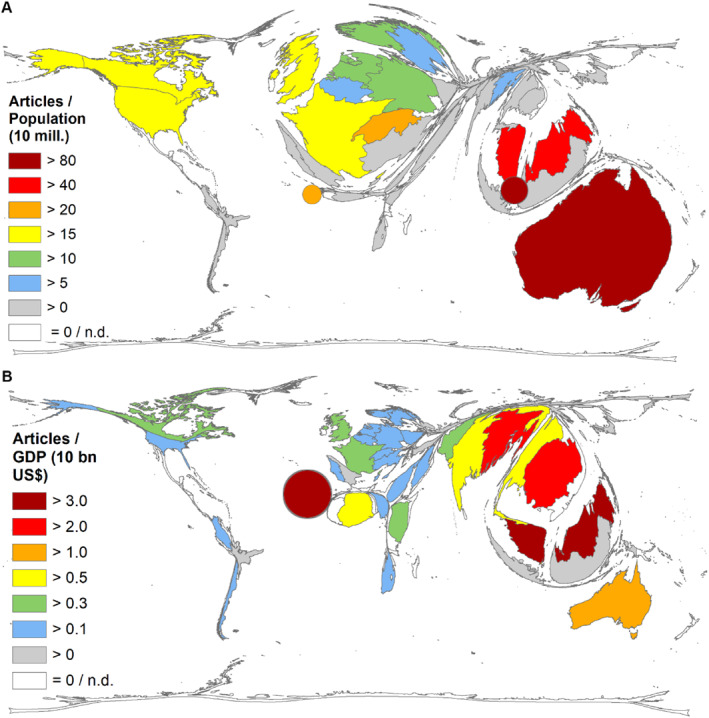
Countries' socio‐economic pattern. (threshold: 5 publications on NiV). (A) *R*_POP_ (Number of articles/population size in 10 mills.). The five countries with the highest *R*_POP_ rates: Singapore, Australia, Malaysia, the Gambia, Switzerland (Singapore and the Gambia are added as points). They could not be shown as an enlarged country due to the (physical limitations of DEMP technology). (B) *R*_GDP_ (Number of articles/gross domestic product (GDP) in 10 bn US). The five countries with the highest *R*_GDP_ rate: The Gambia, Malaysia, Cambodia, Bangladesh, Australia (The Gambia is by far the leader in this analysis. It could not be shown as an enlarged country due to the physical limitations of DEMP technology. It is added as a point).

Regarding the number of citations per country, the picture is roughly the same. Here too, the USA is far ahead with 30,699 citations (c). Australia (*c* = 13,173), Malaysia (*c* = 8298), the UK (*c* = 5044), and France (*c* = 4082) follow next. Only India fell back to 11th place with *c* = 2484. The analyses of the citation rate and the ratios of article numbers to countries' GDP, or population size show different patterns (analysis threshold: *n* = 5):

1. Citation rate (number of citations/number of articles per country) (Figure 3B):
  Ghana (cr = 120.42), Russia (cr = 96.40), South Africa (cr = 80.33), Sweden (cr = 74.00), Singapore (cr = 71.14).
2. *R*_POP_ (number of articles/Population size in 10 million) (Figure 4A):
  Singapore (*R*_POP_ = 71.14), Australia (*R*_POP_ = 58.03), Malaysia (*R*_POP_ = 57.63), The Gambia (*R*_POP_ = 38.71), Switzerland (*R*_POP_ = 28.71).
3. *R*_GDP_ (number of articles/GDP in 10 billion US‐$) (Figure 4B):
  The Gambia (*R*_GDP_ = 33.68), Malaysia (*R*_GDP_ = 3.86), Cambodia (*R*_GDP_ = 2.60), Bangladesh (*R*_GDP_ = 2.33), Australia (*R*_GDP_ = 1.47).

The full lists of all countries with at least five publications on NiV (threshold) are given in Table S1.

**FIGURE 5:**
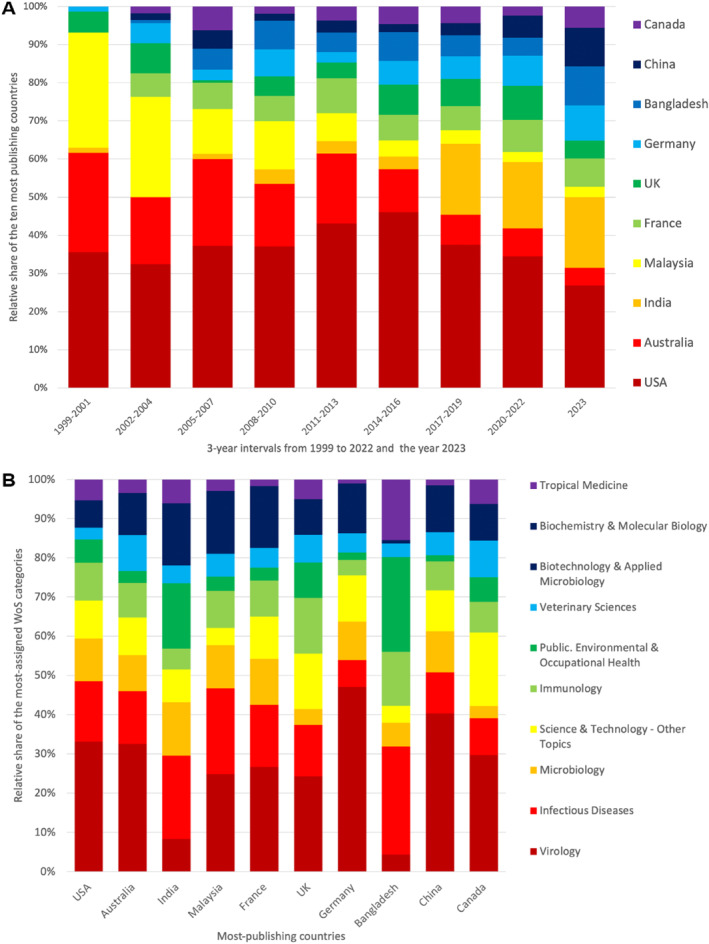
Countries' publication patterns. (A) The relative share of the most‐publishing countries in 3‐year intervals from 1999 to 2022 and 2023. (B) The relative share of the most assigned WoS categories in the most‐publishing countries.

The national contribution to NiV research has changed over time (Figure 5A). The share of Australia and Malaysia decreased between 1999 and 2023, while the share of India and Bangladesh increased. China also expanded its NiV research since 2020. The order of the top 5 in 2023 is therefore as follows: USA (*n* = 29), India (*n* = 20), Bangladesh (*n* = 11), China (*n* = 11), Germany (*n* = 10).

The proportion of the most frequently assigned WoS categories also varies within the national research areas—particularly between high‐income and middle‐ or low‐income economies. For India, Malaysia, and Bangladesh, the research area “Infectious Diseases” plays a major role. Only China, which is also classified as an upper‐middle‐income country, has its focus on “Virology”, as do the high economies among the leading countries (Figure 5B).

### 3.4 Collaborations and Authorships

**FIGURE 6:**
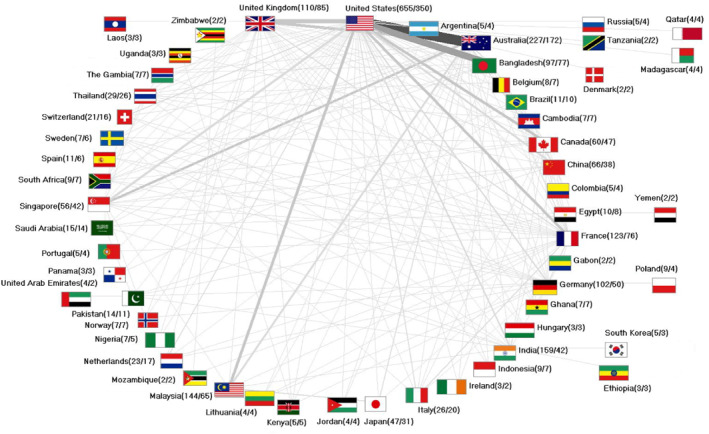
International collaboration network (threshold value for display: 2 collaboration articles). Number in brackets (number of articles/number of collaboration articles). The thickness of the connecting lines indicates the intensity of the collaboration.

A total of *n* = 541 publications (37.80%) were produced in collaboration between at least two countries of origin (*n*_coll_), with binational publications being the most common (*n* = 357; 66% of *n*_coll_). As in the case of all NiV publications (*n*), the annual number has risen over time from *n* = 3 in 1999 to *n* = 40 in 2023. The most productive bilateral cooperation in NiV research took place with the USA. They published the most with Australia (*n* = 112), Bangladesh (*n* = 73), the UK (*n* = 49) and Canada (*n* = 24). Malaysia and France collaborated with the US 30 times each. Researchers from the UK and Canada also collaborated closely (*n* = 32) (Figure 6).

The share of international collaboration in the total number of publications varies from country to country. Of the top 10 countries, Bangladesh (79.38%), Australia (75.77%), and the UK (72.27%) have the highest proportion of *n*_coll_ with partner countries. India (26.42%) cooperates the least with other countries among the top 10. The other top 10 countries lie in between and have a share of 45%–62% of cooperation articles (Table 2).

**TABLE 2:** Collaborations of the 10 most‐publishing countries.

Country	*n*	SA	% SA	*n*_coll_	% *n*_coll_	FA	% FA	CA^a^	% CA^a^
USA	655	305	46.56	350	53.44	151	43.14	168	48.00
Australia	227	55	24.23	172	75.77	66	38.37	80	46.51
India	159	117	73.58	42	26.42	31	73.81	10	23.81
Malaysia	144	79	54.86	65	45.14	21	32.30	40	61.54
France	123	47	38.21	76	61.79	36	47.37	36	47.37
UK	110	25	22.73	85	77.27	28	32.94	40	47.06
Germany	102	52	50.98	50	49.02	15	30.00	31	62.00
Bangladesh	97	20	20.62	77	79.38	52	67.53	25	32.47
China	66	28	42.42	38	57.58	23	60.53	13	34.21

Abbreviations: CA = co‐authorships, FA = first authorships, *n* = number of articles, *n*_coll_ = number of collaboration articles, SA = single authorships.

^a^ The last authorships are included.

In general, it can be seen that countries with lower economic power, such as Thailand, Ghana, Gambia, and Cambodia, have produced all or almost all of their publications in cooperation with other countries (Figure 6).

The proportion of key author positions also varies between countries. India has the highest proportion of first authorships (FAs) among the top ten countries (73.81%), while Germany (30%), the UK (32.94%), and Australia (38.37%) have the lowest proportions (Table 2).

Three multinational scientific partnerships on NiV were written by more than ten countries, with the first authors from Germany, the USA, and Belgium. An article with 13 countries of origin, published in Nature Communications in 2012, dealt with the identification of new paramyxoviruses [40]. Another article, also involving 13 countries, is an article about the Keystone Symposium in 2022, which brought together researchers from academia, government, industry, and non‐governmental organizations to discuss emerging zoonotic viral diseases after the COVID‐19 pandemic. It was published in the Annals of the New York Academy of Science in 2022 [41]. Eleven countries collaborated on a presentation of a disease control tool to secure animal and public health in a densely populated world. It was published in the Lancet Planetary Health in 2022 [42].

**FIGURE 7:**
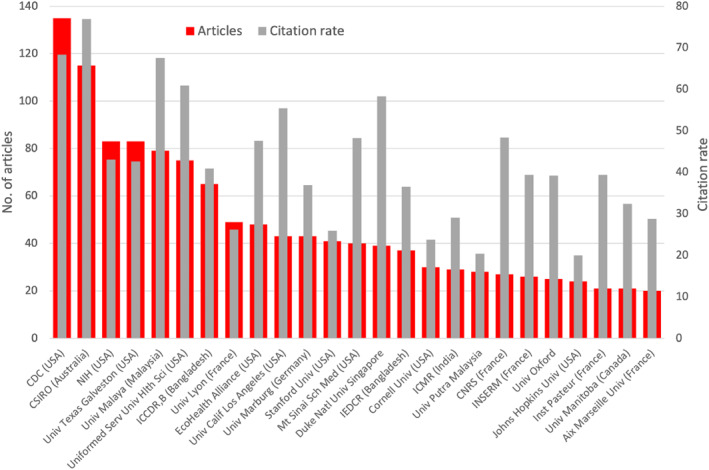
Most publishing institutions (display threshold: 20 articles) with number of articles and citation rate (number of citations/number of articles). CDC = Centre for Disease Control & Prevention, CNRS = Centre National de la Recherche Scientifique, CSIRO = Commonwealth Scientific and Industrial Research Organisation, ICDDR,B = International Centre for Diarrhoeal Disease Research, Bangladesh, ICMR = Indian Council for Medical Research, IEDCR = Institute of Epidemiology Disease Control And Research, INSERM = Institut national de la santé et de la recherche médicale NIH = National Institutes of Health, UCLA = University of California Los Angeles, UTBM = University of Texas Medical Branch, Galveston.

The leading countries also have the most publishing institutions. Two governmental institutions are in the lead. With *n* = 135 articles on NiV, the US Centre for Disease Control & Prevention has published the most articles. It is followed by the CSIRO (Commonwealth Scientific and Industrial Research Organization) from Australia with *n* = 115 articles. The US National Institutes of Health (NIH) is in third place with *n* = 83 together with the University of Texas at Galveston Medical Branch USA (*n* = 83). The University of Malaya in Malaysia is the university with the second most publications (*n* = 79). The Uniformed Service University of Health Science (USA) and the ICDDR,B (International Centre for Diarrhoeal Disease Research, Bangladesh) in Bangladesh follow with *n* = 65 articles each (Figure 7).

The list of the number of articles, citations, and citation rate of all associations with at least 20 articles on NiV is shown in Table S2.

### 3.5 Funding

Of the entire database (*n* = 1431 publications), 91.54% (*n* = 1310) received support, which were listed as funding sources in the article metadata. A total of 2092 grants (*g*) were awarded, corresponding to an average funding of 1.46 grants per publication on NiV. Of these, *g* = 1985 (94.85%) could be assigned to a country of origin. Bilateral or international grants were awarded 79 times, of which the EU (European Union) funded *g* = 46.

In total, 44 countries contributed grants to NiV research, from them 37 governments with *g* = 1538. The governments that provided the most funding were those of the USA (*g* = 737), France (*g* = 131), the UK (*g* = 104), Australia (*g* = 100), and China (*g* = 92). The endemic countries with lower economic power India (*g* = 65), Malaysia (*g* = 39), and Bangladesh (*g* = 13) also contributed research funds for NiV research. The main government organizations funding NiV research were NIH (*National Institutes of Health)* USA (*g* = 535), ANR (*French National Research Agency*) (*g* = 76), UKRI (*UK Research and Innovation)* (*g* = 58), US CDC (*Centre for Disease Control and Prevention*) (*g* = 42), and DFG (*German Research Foundation)* (*g* = 38).

In addition to the governmental funding, universities, scientific institutions, foundations, societies, NGOs, museums, nature parks, and zoos as well as companies and banks have also contributed to the funding of NiV research (Table 3).

**TABLE 3:** Most‐funding countries (threshold: 10 grants).

Country	Gov.	Univ.	RI non‐profit	Found.	Comp./Banks	Soc./Assoc.	NGOs	Parks/Museums
USA	737	127	11	54	13	12	7	6
France	131	6	3	16	4	1	0	0
UK	104	10	0	4	2	6	1	1
Australia	100	3	0	9	4	0	1	1
Germany	76	4	0	15	8	0	1	1
China	92	5	0	1	0	0	0	0
India	65	15	0	2	0	0	0	0
Malaysia	39	28	0	0	0	0	0	1
Canada	37	0	0	0	0	0	0	0
Japan	30	1	0	0	1	0	0	0
Spain	19	0	0	0	2	1	0	0
Sweden	13	3	0	2	0	0	0	0
Bangladesh	13	3	0	0	0	0	0	0
Singapore	15	0	0	0	0	0	0	0
Thailand	14	1	0	0	0	0	0	0
Portugal	10	1	0	2	0	0	0	0
Switzerland	4	3	0	3	2	1	0	0
Italy	3	7	0	1	0	0	0	0
Saudi Arabia	0	11	0	0	0	0	0	0

Abbreviations: Assoc. = Associations, Comp. = companies, Found. = Foundations or trusts, Gov. = governments, NGO = non‐governmental organization, NP = national park, RI = research institutes, Soc. = Societies, Univ. = universities.

**FIGURE 8:**
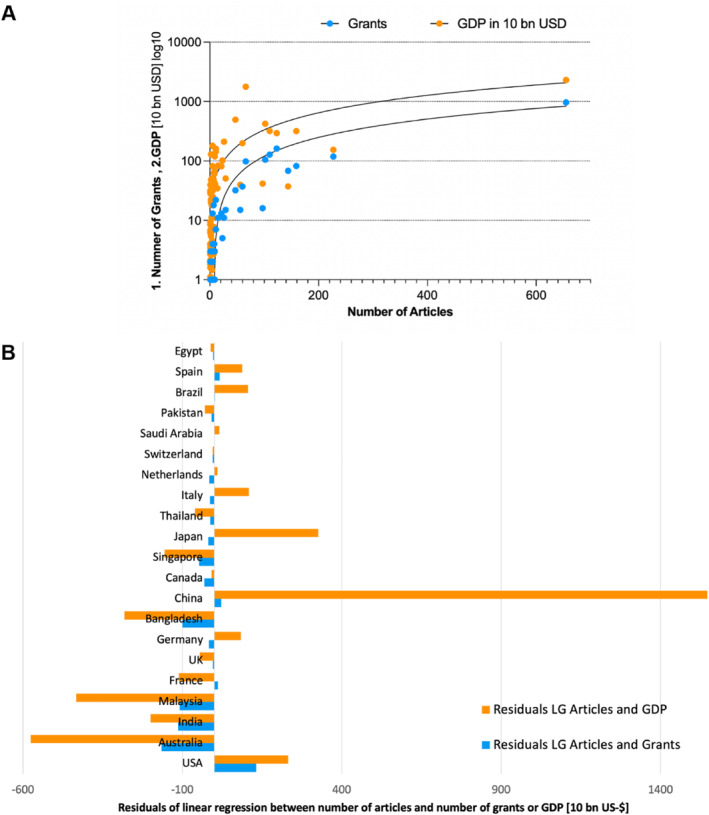
Results of linear Regression. (A) between the number of articles on NiV and the number of grants per country, respectively gross domestic product (GDP) in 10 billion US‐$ per country with at least one article on NiV, logarithmic display. (B) Residuals of countries with at least ten articles on NiV.

The calculation of the linear regression and correlation (Spearman) showed a significant relationship between the number of articles on NiV and the economic power, measured in GDP in US$10 billion (*r* = 0.66, *p* < 0.0001), or the number of granted funds (*r* = 0.69, *p* < 0.0001) of the publishing countries (Figure 8A). The analysis of the respective residuals of the linear regression shows the deviation from the regression line of the publication output of the countries, which can either speak in favour or against the number of articles related to the GDP or the funding for NiV research. That means that in some countries there is a comparatively more committed publication output in relation to the financial possibilities. The USA shows a rather negative deviation for both variables. China shows a strongly negative deviation related to GDP, as was also shown for Japan and Italy. Positive deviations were found for Australia, India, Malaysia, Bangladesh, and Singapore for both variables (Figure 8B).

## 4 Discussion

There is undoubtedly an urgent need for targeted research on NiV globally. Various publication patterns were revealed and discussed in this study to determine the current status and answer the question of what has triggered or hindered research efforts.

As early as 1999, and shortly after NiV was first detected in pig farmers in Malaysia, seven articles were published about it. Among them was one of the most frequently cited articles on fatal encephalitis caused by the infection, published in the journal Lancet [7]. The first author, Kaw Bing Chua, a medical doctor and virologist, was the key scientist during his tenure at the University of Malaya in identifying the etiologic agents that caused this first Malaysian NiV outbreak [43]. He was also the first author of the most frequently cited article from the year 2000 on the classification of NiV as a paramyxovirus and its close relationship to the recently discovered Hendra virus [39]. A third article among the top cited articles was also by Chua et al., dealing with the isolation of NiV from Malaysian Island flying foxes [44]. With three out of ten highly cited articles, Kaw Bing Chua is an outstanding researcher in the field of NiV and has achieved the highest citation rate in this analysis. The annual average citation rate decreases slightly over time in line with the increasing number of articles, with the highest annual value reached in 2020, with only 122 publications. However, the number of annual citations also tends to decline, except for a few peak years. On the one hand, the downward trend in citation numbers and citation rates in recent years is due to the methodological phenomenon of the citation half‐life (CHL) of publications. This refers to the time it takes for an article to reach half of the expected quantity. In the life sciences, the CHL is usually up to 8 years [33]. As far as the NiV trend is concerned, this trend is interrupted by the last peak in 2019. On the other hand, the wave‐like progression of the annual figures is analogous to the outbreak data with peak values in 2001 when cases of NiV infection occurred in India. An almost annual outbreak pattern ‐ with larger outbreaks every 4–5 years ‐ has occurred in Bangladesh and periodically in India since 2001 and explains the likewise undulating publication trend. Of course, the extremely strong and comparatively explosive increase in publications in 2020, the first year of the worldwide COVID‐19 pandemic [45], is understandable due to its global dimension and impact. However, the threat of a new pandemic caused by NiV is indicated by various sources [2] and has suggested a higher level of interest. The flattening trend in NiV interest, which is only briefly interrupted by slight rises following the outbreaks, speaks a different language. In addition to the chronological development, the regional interest in NiV research shows a relatively restrained effort of the otherwise highly engaged high‐income countries. Only the top position of the USA is not unusual, as it is often the main global player in terms of absolute publication figures. However, the following ranks are not occupied by the usually highly engaged European countries, but by Australia, India, and Malaysia, outbreak countries with lower economic power or, in the case of Australia, a high‐income country in the neighbourhood, where the closely related Hendra virus was first described [46].

India, which ranks third in the countries' publication comparison, is one of the most affected countries and the country where the outbreak took place in 2023. The Indian government responded by, among other things, declaring containment zones with corresponding restrictions, as was the case during the COVID‐19 pandemic. Several committees took care of monitoring, testing, training, psychological care, and animal husbandry, among other things. 1288 contacts were recorded in the process [4].

After India, three high‐income EU countries appear in the list of the most publishing countries, directly followed by another low‐income country, Bangladesh. China, which is only in ninth place, is relatively behind in contrast to other research topics, where it has already overtaken the USA [34]. However, China's contribution is increasing over time, so that the country is already in fourth place in 2023. This suggests that it will be one of the leading countries in NiV research in the coming years. Nevertheless, China's contribution to NiV research is comparatively small. No research institution from China is represented in the top group. This is also reflected in the strongly negative deviation of the linear regression residuals between the number of articles and GDP. China has not been affected by NiV outbreaks in the past. Due to the low risk of infection and spillover, the need to promote NiV research was therefore not seen. However, the occurrence of henipavirus‐positive fruit bats and the classification as a risk country by serological and molecular evidence are changing this attitude and increasing the research input [47].

China's research efforts into the SARS‐CoV‐2 virus stand in contrast to this. Here, the first isolation and case of COVID‐19 occurred in Wuhan City, China [48]. Analyses of the publication output on the Mpox virus, for example, show that the annual numbers also remain in double digits and increased only slightly when the virus first reached the USA. However, due to the outbreak in 2022, the number increased extraordinarily, reaching more than 400 articles, similar to the much larger, explosive increase in coronavirus studies in 2020, which rose to more than 30,000 [49]. Corona research was shown to have a brief interlude during the emergence of SARS (Severe Acute Respiratory Syndrome), similar to MERS (Middle East Respiratory Syndrome), which was also of only brief interest in the scientific community [45].

During the COVID‐19 pandemic, it became clear how vulnerable national health systems are to novel pathogens [31] and how quickly viral infections develop into serious global problems [50]. This illustrates the prioritisation of other high‐risk pathogens, such as NiV, in surveillance and preparedness programs and early warning systems. A rapid response to a pandemic requires extensive international cooperation and coordination, for example, in the implementation of management and surveillance plans for wildlife such as bat populations and in the training of local health experts [51, 52]. Research into vaccines and therapeutics is also essential but also requires better international cooperation and stable funding.

Although the experience of the COVID pandemic has led to improvements, global inequality is evident as low‐ and middle‐income countries are nowhere near as well‐resourced as high‐income countries [53].

As far as NiV research is concerned, it is clear that the highly affected countries are in first place when calculating the relationship between the number of articles and the countries' GDP. It could also be shown that this research topic is characterised by a significantly higher performance of the affected low economies [34]. However, the proportion of international cooperation articles varies between the countries. The Bangladeshi articles, for example, were mostly written in almost 80% international collaboration with the USA, while the Malaysian ones collaborated significantly less internationally at around 45% and India only around 26%. On the other hand, the proportion of first authorships in Malaysian articles is significantly lower than in those of other outbreak countries. India, in particular, is very high in this respect, with more than 73% first‐authorships of their collaborations. These characteristics show the high participation and responsible authorship positions of affected countries. However, this must be seen in relation to the low number of global articles on NiV. These facts make it clear that the interest of large economies with good scientific infrastructure sets the limits of NiV research. As NiV is classified as a category‐4 virus, a BSL‐4 laboratory is required to handle this virus. In particular, the limited access in Asia, with currently only nine BSL‐4 laboratories, makes international cooperation with the countries concerned necessary. The planning of BSL‐4 laboratories and the associated transfer of expertise, particularly in Asian countries such as India, is a step in the right direction [25]. These laboratories are crucial for control and surveillance. They determine the microbial cause of disease syndromes and detect and report pathogens. They also assess antimicrobial resistance. To this end, they must be safe, well‐equipped facilities that are subject to strict control criteria, for which intensive networking is important [54].

The risk of NiV spreading beyond the still regionally limited confines is given by globalisation and worldwide travel opportunities. In particular, neighbouring countries with bat species known to be infected with NiV need to be prepared. The international development of concepts with stakeholders from the affected regions must be incorporated into research measures. Taking into account acceptance and trust barriers in the affected population, scientifically sound, culturally and socio‐economically viable solutions can be found. This should include free, easily accessible public healthcare to protect entire families or communities [55]. In addition, local institutions must be involved and financially supported to achieve a long‐term impact through equitable measures [32]. If necessary, measures to restrict trade are justified [5].

Priority should be given to public education and screening, surveillance initiatives, and the development of response plans that include international cooperation in resource allocation and information sharing. Targeted joint scientific research on pathogenicity, epidemiology, pest control, intervention, and prevention is also essential [56, 57].

## 5 Conclusions

The comparatively low scientific engagement of countries that are usually key players in global scientific publications and the prevailing lack of knowledge in NiV infectiology combined with the threat of NiV spreading to other areas are exceedingly threatening.

The results of this study may show parallels to the global engagement in coronavirus or Mpox virus research, which was also temporarily stimulated by regional outbreaks, followed by lower interest. In consequence, in the case of coronavirus research, knowledge gaps still exist, which certainly stood in the way of developing suitable countermeasures during the global COVID‐19 pandemic. As is often said, the NiV infection belt is small but deadly, but global travel and trade increase the risk of spread. The global scientific community must be prepared for the possible spread of infections that pose a pandemic risk. Especially in the absence of a vaccine, awareness, education, and training are crucial. Easy and free access to healthcare with safe protocols is essential. This, as well as the development and maintenance of sustainable integrated surveillance programs, also requires stable funding and a solid scientific basis that benefits from international cooperation.

## Author Contributions

**Doris Klingelhöfer:** conceptualization, data curation, investigation, methodology, formal analysis, supervision, visualisation, writing – original draft, writing – review and editing. **Markus Braun:** resources, validation, visualisation, writing – review and editing. **Christina A. Naser:** resources, writing – review and editing. **Dörthe Brüggmann:** resources, validation, visualisation, writing – review and editing. **David A. Groneberg:** project administration, resources, software, writing – review and editing.

## Ethics Statement

The authors have nothing to report.

## Consent

The authors have nothing to report.

## Conflicts of Interest

The authors declare no conflicts of interest.

## Supporting information

Supporting Information S1

## Data Availability

Underlying data is available upon reasonable request.
