# Supplementary material for: Emerging Nipah Virus With Pandemic Potential and High Mortality Rates: Is the Scientific Community Learning From Former Pandemics?
Source: Rev Med Virol. 2025 Mar 5;35(2):e70028. doi: 10.1002/rmv.70028 (PMC11882410; doi:10.1002/rmv.70028)
Supplement: Supplementary file 1 — Supporting Information S1 [file RMV-35-e70028-s001.docx]

**Supplement**

**Supplementary tables**

Supplementary Table 1: Socio-economic ratios between the number of articles on NiV and the GDP (gross domestic product) in 10 billion US-Dollars (R_GDP_) and the population in 10 million inhabitants (R_POP_) for countries with at least five articles on NiV (threshold), sorted by R_GDP_. Countries’ classification of the World Bank:^1^ HI = high-income country, UMI = upper-middle-income country, LMI = lower-middle-income country, LI = low-income country.

| **Country** | **Articles** | **GDP** [10 bn USD] | **R_GDP_** | **Rank R_GDP_** | **Population** [10 mill.] | **R_POP_** | **Rank  R_POP_** |
| --- | --- | --- | --- | --- | --- | --- | --- |
| The Gambia | 7 | 0.21 | 33.69 | LI 1 | 0.25 | 28.15 | LI 1 |
| Malaysia | 144 | 37.27 | 3.86 | UMI 1 | 3.28 | 43.93 | UMI 1 |
| Cambodia | 7 | 2.70 | 2.60 | LMI 1 | 1.69 | 4.13 | LMI 2 |
| Bangladesh | 97 | 41.63 | 2.33 | LMI 2 | 16.63 | 5.83 | LMI 1 |
| Australia | 227 | 154.27 | 1.47 | HI 1 | 2.58 | 88.03 | HI 2 |
| Singapore | 56 | 39.70 | 1.41 | HI 2 | 0.55 | 102.70 | HI 1 |
| Ghana | 7 | 7.76 | 0.90 | LMI 3 | 3.17 | 2.21 | LMI 3 |
| Thailand | 29 | 50.60 | 0.57 | UMI 2 | 7.00 | 4.15 | UMI 2 |
| India | 159 | 317.34 | 0.50 | LMI 4 | 139.34 | 1.14 | LMI 4 |
| Kenya | 5 | 11.03 | 0.45 | LMI 5 | 5.50 | 0.91 | LMI 6 |
| France | 123 | 293.75 | 0.42 | HI 3 | 6.75 | 18.22 | HI 5 |
| Pakistan | 14 | 34.63 | 0.40 | LMI 6 | 22.52 | 0.62 | LMI 7 |
| UK | 110 | 318.69 | 0.35 | HI 4 | 6.82 | 16.13 | HI 6 |
| Canada | 60 | 199.08 | 0.30 | HI 5 | 3.81 | 15.76 | HI 7 |
| USA | 655 | 2299.61 | 0.28 | HI 6 | 33.19 | 19.74 | HI 4 |
| Switzerland | 21 | 81.29 | 0.26 | HI 7 | 0.87 | 24.10 | HI 3 |
| Egypt | 10 | 40.41 | 0.25 | LMI 7 | 10.43 | 0.96 | LMI 5 |
| Germany | 102 | 422.31 | 0.24 | HI 8 | 8.39 | 12.16 | HI 10 |
| Netherlands | 23 | 101.80 | 0.23 | HI 9 | 1.72 | 13.39 | HI 8 |
| South Africa | 9 | 41.99 | 0.21 | UMI 3 | 6.00 | 1.50 | UMI 3 |
| Portugal | 5 | 24.99 | 0.20 | HI 10 | 1.02 | 4.92 | HI 13 |
| Saudi Arabia | 15 | 83.35 | 0.18 | HI 11 | 3.53 | 4.24 | HI 15 |
| Colombia | 5 | 31.43 | 0.16 | UMI 4 | 5.13 | 0.98 | UMI 4 |
| Nigeria | 7 | 44.08 | 0.16 | LMI 8 | 21.14 | 0.33 | LMI 9 |
| Norway | 7 | 48.24 | 0.15 | HI 12 | 0.55 | 12.81 | HI 9 |
| Poland | 9 | 67.40 | 0.13 | HI 13 | 3.78 | 2.38 | HI 17 |
| Belgium | 8 | 59.99 | 0.13 | HI 14 | 1.16 | 6.88 | HI 12 |
| Italy | 26 | 209.99 | 0.12 | HI 15 | 6.04 | 4.31 | HI 14 |
| Sweden | 7 | 62.74 | 0.11 | HI 16 | 1.02 | 6.89 | HI 11 |
| Argentina | 5 | 49.15 | 0.10 | UMI 5 | 4.56 | 1.10 | UMI 5 |
| Japan | 47 | 493.74 | 0.10 | HI 17 | 12.57 | 3.74 | HI 16 |
| Spain | 11 | 142.53 | 0.08 | HI 18 | 4.67 | 2.35 | HI 18 |
| Indonesia | 9 | 118.61 | 0.08 | UMI 6 | 27.64 | 0.33 | UMI 10 |
| Brazil | 11 | 160.90 | 0.07 | UMI 7 | 21.40 | 0.51 | UMI 7 |
| Türkiye | 5 | 81.53 | 0.06 | UMI 8 | 8.50 | 0.59 | UMI 6 |
| China | 66 | 1773.41 | 0.04 | UMI 9 | 144.42 | 0.46 | UMI 8 |
| Russia | 5 | 177.58 | 0.03 | UMI 10 | 14.59 | 0.34 | UMI 9 |
| South Korea | 5 | 179.85 | 0.03 | HI 19 | 5.13 | 0.97 | HI 19 |
| Iran | 5 | n.a. | n.a. | LMI | 8.50 | 0.59 | LMI 8 |

Supplementary Table 2: Most-publishing institutions with at least 20 articles on NiV.

*Abbreviations: CDC = Center for Disease Control & Prevention, CSIRO = Commonwealth Scientific and Industrial Research Organisation, NIH = National Institutes of Health, UTBM = University of Texas Medical Branch, Galveston, ICDDR,B = International Centre for Diarrhoeal Disease Research, Bangladesh, UCLA = University of California Los Angeles, IEDCR = Institute of Epidemiology Disease Control And Research, ICMR = Indian Council for Medical Research, CNRS = Centre National de la Recherche Scientifique, INSERM = Institut national de la santé et de la recherche médicale.*

| **Institution** | **Country** | **Articles** | **Citations** | **Citation rate** |
| --- | --- | --- | --- | --- |
| US CDC | USA | 135 | 9227 | 68,35 |
| CSIRO | Australia | 115 | 8849 | 76,95 |
| NIH | USA | 83 | 3575 | 43,07 |
| UTMB | USA | 83 | 3534 | 42,58 |
| University of Malaya | Malaysia | 79 | 5336 | 67,54 |
| Uniformed Service University | USA | 75 | 4571 | 60,95 |
| ICDDR,B | Bangladesh | 65 | 2658 | 40,89 |
| University Lyon | France | 49 | 1282 | 26,16 |
| EcoHealth Alliance | USA | 48 | 2282 | 47,54 |
| University of Marburg | Germany | 43 | 1587 | 36,91 |
| UCLA | USA | 43 | 2382 | 55,40 |
| Stanford University | USA | 41 | 1063 | 25,93 |
| Mt Sinai School of Medicine | USA | 40 | 1928 | 48,20 |
| Duke National University of Singapore | Singapore | 39 | 2274 | 58,31 |
| IEDCR | Bangladesh | 37 | 1351 | 36,51 |
| Cornell University | USA | 30 | 712 | 23,73 |
| ICMR | India | 29 | 843 | 29,07 |
| University Putra Malaysia | Malaysia | 28 | 570 | 20,36 |
| CNRS | France | 27 | 1306 | 48,37 |
| INSERM | France | 26 | 1023 | 39,35 |
| University of Oxford | UK | 25 | 980 | 39,20 |
| Johns Hopkins Bloomberg School of Public Health | USA | 24 | 478 | 19,92 |
| University of Manitoba | Canada | 21 | 679 | 32,33 |
| Institut Pasteur | France | 21 | 826 | 39,33 |
| Aix Marseille University | France | 20 | 575 | 28,75 |

**References**

1. World_Bank. World Bank Country and Lending Group, URL: <https://datahelpdesk.worldbank.org/knowledgebase/articles/906519-world-bank-country-and-lending-groups> (accessed Feb 2024). 2022.
